# Optimal nutrition and the ever-changing dietary landscape: a conference report

**DOI:** 10.1007/s00394-017-1460-9

**Published:** 2017-05-05

**Authors:** A. Shao, A. Drewnowski, D. C. Willcox, L. Krämer, C. Lausted, M. Eggersdorfer, J. Mathers, J. D. Bell, R. K. Randolph, R. Witkamp, J. C. Griffiths

**Affiliations:** 1 Herbalife Nutrition, Los Angeles, CA USA; 2 University of Washington, Seattle, WA USA; 3 Okinawa International University, Ginowan, Japan; 4 Technische Universität Braunschweig, Brunswick, Germany; 5 Institute for Systems Biology, Seattle, WA USA; 6 DSM Nutritional Products, Kaiseraugst, Switzerland; 7 Newcastle University, Newcastle upon Tyne, UK; 8 University of Westminster, London, UK; 9 Amway Global Discovery, Buena Park, CA USA; 10 Wageningen University, Wageningen, The Netherlands; 11 Council for Responsible Nutrition-International, Washington, DC USA

**Keywords:** Aging, Big data, Bioactives, Biomarkers, Dietary patterns, Dietary supplements, Longevity, Micronutrients, Obesity, Overfed, Phytonutrients, Sarcopenic obesity, Systems approaches, Undernourished, Wellness

## Abstract

The field of nutrition has evolved rapidly over the past century. Nutrition scientists and policy makers in the developed world have shifted the focus of their efforts from dealing with diseases of overt nutrient deficiency to a new paradigm aimed at coping with conditions of excess—calories, sedentary lifestyles and stress. Advances in nutrition science, technology and manufacturing have largely eradicated nutrient deficiency diseases, while simultaneously facing the growing challenges of obesity, non-communicable diseases and aging. Nutrition research has gone through a necessary evolution, starting with a reductionist approach, driven by an ambition to understand the mechanisms responsible for the effects of individual nutrients at the cellular and molecular levels. This approach has appropriately expanded in recent years to become more holistic with the aim of understanding the role of nutrition in the broader context of dietary patterns. Ultimately, this approach will culminate in a full understanding of the dietary landscape—a web of interactions between nutritional, dietary, social, behavioral and environmental factors—and how it impacts health maintenance and promotion.

## From reductionism to holism: the evolution of nutrition science

Compared to the classical natural sciences (chemistry, physics and mathematics) nutrition science is a relatively new discipline. In the early twentieth century, the public health challenges around nutrition mainly focused on communicable diseases and overt nutrient deficiencies, at the time major determinants of a short life expectancy. To address these concerns, the scientific focus of the time was devoted to vitamins and essential minerals to combat the effects of malnutrition.

During this time, scientists believed that single nutrients could cure or reverse particular diseases. This concept was readily accepted by the scientific and medical communities because it was simple and based on three premises [95]:

1. A simple cause–effect relationship exists between a particular nutrient and a specific effect or disease.
2. Symptoms of a specific nutrient deficiency can be physiologically explained in terms of the role played by the respective nutrient.
3. Providing the nutrient in the diet can prevent, and in many cases reverse, the deficiency disease.

This paradigm was appropriate for addressing the then challenges of overt nutrient deficiency. However, through the course of the twentieth century the situation changed rapidly, especially in developing nations. Public health and nutrition challenges, which had been rooted in deficiency, shifted to challenges resulting from excess. By the mid-to-late twentieth century, major public health challenges transitioned from communicable diseases and malnutrition as the major cause of mortality, to lifestyle-related chronic diseases [7]. These contemporary challenges are due to a combination of poor diet, reduced physical activity, rapidly aging population, rapidly expanding population, climate change and food security issues. The reductionist research model has proven to be ill-suited to address these challenges.

In response to the growing challenges of non-communicable diseases, public and private investments in science and research grew dramatically and have achieved some important beneficial milestones.

- Advances in understanding the role of diet and nutrition in the etiology of chronic diseases.
- Advances in cellular and molecular biology, and biochemistry to allow for a better understanding of macro and micronutrient metabolism and mechanisms of action.
- Advances in identification of nutrients linked to chronic disease; for example, calcium, folate, vitamin D, omega-3 fatty acids and dietary fiber.
- Advances in the discovery, study, and use of other bioactive substances found in foods, such as isoflavones, carotenoids, anthocyanins and catechins.
- Advances in the understanding of the impact of the microbiome on immunity, obesity and cognitive function.
- Advances in the various ‘omics’ technologies: genomics, transcriptomics, proteomics and metabolomics, along with epigenetics.

However, despite these advances, from a public health perspective, the emphasis on a healthy diet and lifestyle has not had the anticipated impact. Imbalanced diet and poor lifestyle have emerged as major contributors to early death [128] coinciding with a global obesity epidemic [46] and proliferation of co-morbidities such as type II diabetes [47]. The nutrition imbalance, where nutrient adequacy is worsening while energy (calorie) excess continues to rise, has contributed to a paradox of obesity combined with undernutrition. Populations have become overfed, but undernourished [78].

In the 1980s and 1990s, nutrition science entered an era during which the ‘magic bullet’ was sought using a reductionist approach. This has been referred to as ‘greedy reductionism’:

…in their eagerness for a bargain, in their zeal to explain too much too fast, scientists and philosophers… underestimate the complexities, trying to skip whole layers or levels of theory in their rush to fasten everything securely and neatly to the foundation [30].

Nutrients were included in drug-like randomized controlled trials, the most notable were the studies in which vitamin E and/or *beta*-carotene were given to lifelong smokers or asbestos workers, with the ill-conceived hypothesis that these nutrients could reverse decades of smoking or asbestos exposure [90, 107]. In an effort to uncover the magic bullet, scientists inappropriately studied nutrients in a drug-like context. Unlike drugs, nutrients do not function in isolation and have beneficial effects on multiple tissues and organ systems; a narrow focus on a single or ‘primary’ outcome measure is not practical and does not fit the nutritional context [50].

Perpetuated by a reductionist approach on single macro- and micronutrients, scientists have similarly spent countless resources satisfying the demand for the nutrition ‘villain’ and ‘hero.’ Heroes include substances such as anti-oxidants, fiber, protein and probiotics, while the maligned villains include saturated fat, refined carbohydrates, trans-fat, salt and sugar. In studying the effects of nutrients in isolation, premature conclusions have been reached, resulting in the inevitable ‘flip-flopping’ on whether particular nutrients are beneficial or harmful. This, in turn, has led to enormous consumer confusion and frustration. The problem with this reductionist approach is that, in emphasizing specific nutrients, it fails to take into account that food components interact in complex ways to give rise to emergent properties of diets that are not explicable at the level of individual chemical parts.

During that time span, the perspective of nutrition research has changed from a reductionist approach focusing on specific nutrients, to a complex systems-based science domain. According to the definition of nutrition science for the twenty-first century from *The Giessen Declaration,* an important international workshop sponsored by the International Union of Nutritional Sciences and the World Health Policy Forum: ‘Nutrition science is defined as the study of food systems, foods and drinks, and their nutrients and other constituents; and of their interactions within and between all relevant biological, social and environmental systems’ [108].

Nutrition research is now described as a field which integrates a variety of disciplines, including biology, physiology, sociology, economics, politics and environment [19]. Researchers are now calling for the consideration of environmental sustainability when addressing nutrition questions of public health significance [5]. To grasp these dynamic and highly intertwined connections and interactions, a systems approach is now being advocated, which incorporates transdisciplinary models for food and nutrition security. Such an approach aims to reduce complexity by revealing key interactions that influence the outcomes of interest [48]. In other words, a systems approach can help to identify common causal factors underlying the otherwise seemingly opposing challenges of malnutrition and obesity (population is undernourished, but overfed), which are occurring with more regularity around the world.

The recognition of the need for an interdisciplinary approach to nutrition research has fortunately made its way to the Federal research agenda in the United States. The *National Nutrition Research Roadmap* released by the Interagency Committee on Human Nutrition Research [56] includes highly relevant research questions targeting areas such as behavioral, lifestyle, social, cultural, economic, occupational and environmental factors that impact food choices. The report highlights the importance of environmental sustainability and social science as it pertains to maintaining healthy eating patterns.

Technology now allows use of ‘small data’ at the individual level to drive personalization of diets [129]. Handheld devices now assist consumers allowing them to understand their own nutrient and health status and to make choices. Biomarkers of nutrition status are replacing intake assessment as the basis for identifying dietary gaps. Recommendations for healthy dietary patterns are complimenting, and in some cases replacing those for specific foods and nutrients [111]. These advancements are part of the evolution from linking health benefits to specific nutrients at specific doses to understanding the broader dietary landscape that impacts health including food policy, food choices, culture, environment, dietary patterns, social/psychological factors, home/workplace/school and economy.

Despite a number of challenges and setbacks, nutrition science has developed significantly over time (and will continue). The scientific focus of nutrition had narrowed with a reductionist approach and subsequently (now) expanded to be more holistic. It is now recognized that the study of nutrition involves more than the biology of nutrients, but encompasses the integration of other scientific disciplines, including social, political and environmental sciences. Nutrition recommendations and policy need to continue to evolve in parallel with advances in science and technology to provide solutions to contemporary public health challenges.

## Undernourished but overfed: the social and economic considerations

Global diets have become rich in calories but can be poor in some essential nutrients. More and more people are becoming overweight and obese while failing to meet dietary nutrient requirements. As a result, overweight and nutrient deficiencies, the latter sometimes known as ‘hidden hunger’, are both viewed as different forms of malnutrition.

Ideally, each food calorie ought to be the dietary vector for essential micronutrients, e.g., vitamins and minerals. It was long assumed that dietary nutrients and calories went together. However, that relation has now been uncoupled by the emergence in the modern food supply of low-cost empty calories from refined grains, added sugars and added fats. If low-cost fats and sweets provide dietary energy but no nutrients, then eating more food provides no further nutritional benefits. The link between energy consumption and nutrient intake is broken. The low cost of empty calories has made it possible to become undernourished but overfed.

Dietary advice has not kept pace with the basic economics of food-choice behavior. The Dietary Guidelines for Americans 2015–2020 have restated the traditional position that all nutritional needs should be met primarily from nutrient-dense foods. The recognized components of healthy eating patterns were fresh vegetables, legumes, whole fruits whole grains, fat-free or low-fat dairy, seafood, lean meats and poultry, as well as nuts and seeds. Other expert panels have gone further in recommending that all dietary nutrients should come from wholesome ‘real’ foods, preferably local, fresh, unprocessed, and organic. The 2015 Dietary Guidelines did allow that, in some cases, fortified foods and dietary supplements could be useful in providing selected nutrients that might otherwise be consumed in less than recommended amounts [111].

Underconsumed nutrients of greatest public health concern were identified as calcium, potassium, dietary fiber and vitamin D. Iron, underconsumed by adolescent girls and adult women, was another nutrient of public health concern. The suggested remedy was to shift to eating patterns with more vegetables, fruits, whole grains and dairy. Vegetables, fruit juices and dairy are important sources of potassium; vegetables and whole grains supply fiber; whereas dairy is the principal source of both calcium and vitamin D.

Although sustainable food production was an issue, the wide disparities in food prices, seemingly, were not. The nutritive value of foods is often related to their per calorie cost [33, 34]. Among the chief food sources of potassium, the Dietary Guidelines listed low-cost baked potato but also wild Atlantic salmon, Swiss chard and pomegranate juice. Among fiber sources were a wide variety of beans and ready-to-eat cereals but also avocados, artichokes and raspberries. Calcium was provided by milk and cheese and by various fortified ready-to-eat cereals. Only pregnant women were advised to take an iron supplement when recommended by a healthcare provider. Other supplements were not mentioned.

Yet, it is food prices and diet costs that mediate the observed links between poverty, obesity and micronutrient deficiencies [26, 80]. The following arguments can be made.

First, the current hierarchy of food prices is such that energy-dense refined grains, added sugars, and vegetable oils have become the least expensive sources of concentrated dietary energy, as measured in calorie per unit cost [33, 34]. Total diets composed of energy-dense foods tend to be cheaper per calorie than low energy density diets built around the recommended healthier options [26]. In such studies, food energy density was measured in terms of kcal/100 g, whereas dietary energy density was measured in terms of cost per 1800 or 2000 kcal.

**Fig. 1:**
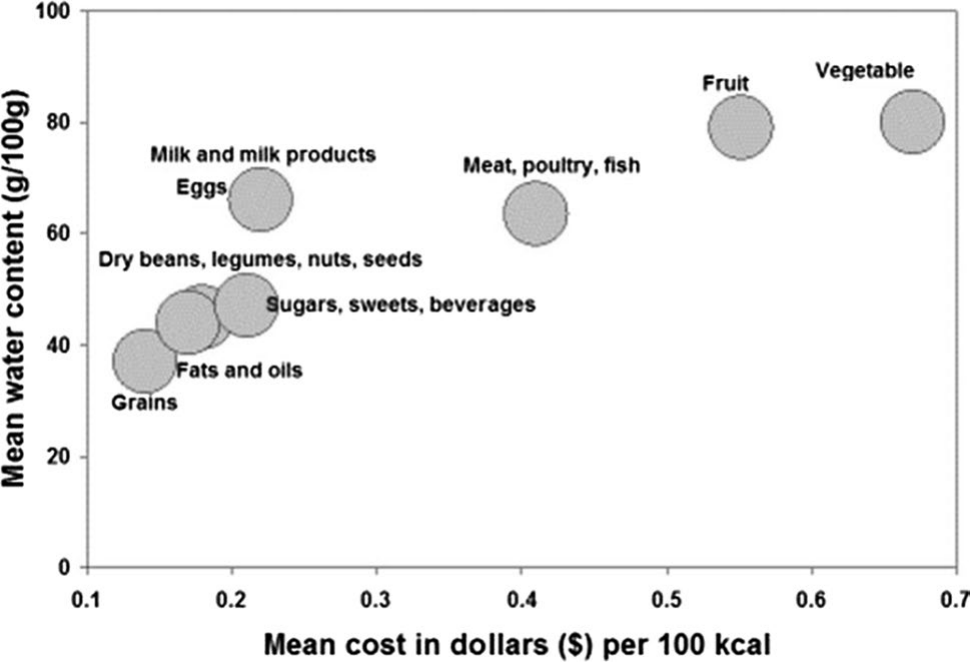
Relation between mean cost per 100 kcal in US dollars and water content of foods in g/100 g. Data are for 1387 foods from the USDA Food and Nutrient Database for Dietary studies (FNDDS 2.0) aggregated by major food groups

As shown in Fig. 1, energy density of foods is determined by their moisture content. This is because water provided bulk and weight but no energy and no nutrients. In general, energy-dense foods are foods that are dry. Those foods are associated with lower per calorie costs. Not by coincidence, grains, fats and sweets provided calories at low cost, as did sugar sweetened beverages.

By contrast, the more nutrient-rich meat, poultry, fish, vegetables and fruit were more expensive. Nutrient density of foods has been measured in terms of nutrients per 100 kcal, 100 g or serving size. Typically, nutrient-rich foods are those that contain more nutrients than calories. The so-called qualifying nutrients have included protein, fiber, and a variety of vitamins and minerals, including those of public-health concern. Disqualifying nutrients have typically included fat, sugar and sodium. Nutrient profiling is the technique used to separate foods that are energy-dense from those that are nutrient-rich [32, 35]. Nutrient profiling has also been used to assess nutrients per unit cost and the environmental cost of nutrient production.

The more nutrient-rich foods not only did cost more per calorie, but the price disparity between energy-dense and nutrient-rich foods has continued to grow. Tracking the cost of a market basket of 384 foods from 2004 to 2016 has shown that prices have increased the most for meat, poultry and seafood, fresh produce and whole fruit. Whereas, processed fats and sweets cost only 30% more than they did 20 years ago; the cost of fresh produce has more than doubled. As might be expected, energy-dense processed foods, including many grain-based desserts, provide palatable calories at low cost.

The shift to cheap energy-dense foods can lead to overeating due to diminished satiety signals. Whereas, vegetables and whole fruit can be bulky but low in energy, dry foods provide concentrated energy in small volume. Whereas, carrots provide 40 kcal/100, the energy density of a bar of chocolate can be several times higher, around 500 kcal/100 g. In multiple clinical studies, energy-dense foods and energy-dense diets have been linked to overeating and weight gain.

Thus, the observed links between poverty and obesity may be mediated, in part, by the low cost of energy-dense foods and reinforced by the high palatability of sugar and fat. In the US, population subgroups with less education and lower incomes had lower food expenditures and lower quality diets. Those groups were least likely to consume the recommended nutrient-rich foods and were more likely to suffer from micronutrient deficiencies, notably for the nutrients of public health concern. It has been well documented elsewhere that groups of lower socioeconomic status were also more likely to be obese [26].

As a result, populations in developed countries suffer from the dual burden of malnutrition: micronutrient deficiencies and overweight. Both conditions are linked to socioeconomic disparities and are most prevalent among population subgroups with low economic resources and high poverty rates. Improving the nutrient-to-calorie ratio, rather than focusing on calories alone, may be an effective strategy for obesity prevention and control.

## Dietary patterns and longevity: insights from the oldest old in Okinawa

The proportion of worldwide mortality from chronic age-associated disease is projected to reach two-thirds of all deaths by 2030 [126]. This global increase in disease burden from cardiovascular disease (CVD), cancer, diabetes and other chronic age-associated diseases reflects an aging population, as well as social and economic changes, including lifestyle changes and a global nutrition transition to dietary patterns that are more energy-dense but poor in some essential nutrients (see “Undernourished but overfed: the social and economic considerations”, this article). Despite these challenges, effective public health policy and program implementation can do much to mitigate this risk and help people remain healthy as they age.

Reflecting the potential for preventive public health efforts, the World Health Organization (WHO) has estimated that 80% of coronary heart diseases and type-2 diabetes, as well as 40% of cancers could be prevented by better management of three health behaviors: dietary habits, physical activity and the use of tobacco products [126]. Of these three risk factors, dietary habits may have become the most important modifiable risk factor in many nations, as suggested by a recent study in the United States, that assessed 17 major risk factors. Composition of the diet made up the largest cluster of risk factors responsible for death (26%) and the highest percentage of disability-adjusted life years lost at 14% [83].

Because nutritional issues play such a key role in a wide range of age-associated diseases, the potential for better dietary habits to improve health outcomes in aging populations is great. The Okinawans are of special interest to this topic, as they have been celebrated as the longest lived of the Japanese since prefectural data became available, and nutritional factors may have played a key role in this healthy aging phenomenon, with low rates of cardiovascular disease (and some cancers) mirroring WHO estimates of what may be potentially achieved by preventive public health efforts [105].

Much of the longevity advantage in Okinawa is thought to be related to a healthy lifestyle and this includes the traditional diet, which is calorie-poor, yet nutritionally dense, particularly with regard to phytonutrients with potential caloric restriction (CR) mimetic properties [122]. Although many similarities exist between the traditional Okinawan and Japanese dietary patterns, including the high intake of vegetables; the abundance of soy products such as tofu, natto and miso; the low intake of fats and oils; the heavy reliance on seafood and seaweeds; and the minimal consumption of dairy products, the traditional Okinawan diet differs in some key areas. In particular, the staple food of the Okinawan diet was neither polished white rice nor other grains but many varieties of colorful sweet potatoes.

Okinawan sweet potatoes have several interesting nutritional characteristics that may have anti-aging effects. For example, sporamin comprises most of the total protein in the edible tuberous root. The biological functions of sporamin include strong free-radical inhibitory and scavenging activity [99]. In light of the strong connection between inflammation and aging, or ‘inflammaging’, as termed by Franceschi and colleagues [40], it is of particular interest that the sweet potato has significant anti-inflammatory properties from multiple phytonutrients that include phenolic acids, flavonols, anthocyanins and carotenoids [124]. In addition to its strong anti-inflammatory effects, sweet potatoes are also good sources of B vitamins, including folate, thiamine, riboflavin, and vitamin B6. Folate and vitamin B6 help convert homocysteine into harmless cysteine. Since high homocysteine levels are associated with an increased risk of cardiovascular disease, it is also of considerable interest that low homocysteine levels accompany the low CVD mortality in Okinawa [4].

Once regarded derisively as a ‘poor farmer’s food’ by those in the upper classes, sweet potatoes now are highly recommended for their health-enhancing properties with recommendations coming from the American Heart Association, the American Cancer Society, as well as the Center for Science in the Public Interest (CSPI) [122]. In fact, the CSPI recently ranked the sweet potato as the ‘healthiest of all vegetables’, mainly for its high content of dietary fiber and protein, anti-oxidant vitamins A and C, potassium, iron, calcium and low levels of saturated fat, sodium and cholesterol [122]. Moreover, most of these nutrients are the very same that are lacking in the American (or ‘Westernized’) diet.

Traditional Okinawan cuisine revolves around steamed sweet potatoes, simmered or steamed green leafy and/or yellow root vegetables, and soy (e.g., miso soup, tofu, miso flavorings), which accompanies almost every meal. Smaller servings of fish or lean meats flavored with herbs and spices, often accompany these staples [124]. Other characteristics that define the Okinawan diet include a taste for bonito flavored broths, and the liberal use of herbs and spices in place of salt.

Ten characteristics of the traditional Okinawa diet:

1. low-caloric intake,
2. high-vegetable consumption (particularly yellow root and green leafy vegetables and seaweeds),
3. high consumption of legumes (mostly soybean),
4. moderate consumption of seafood (more in coastal areas),
5. low consumption of meat (mostly lean pork),
6. little to no dairy products,
7. low-fat intake (high mono and polyunsaturated/saturated fat ratio),
8. low-glycemic load,
9. high-fiber intake,
10. moderate alcohol consumption.

Research suggests that dietary patterns associated with a reduced risk of chronic age-associated disease are vegetable and fruit heavy (therefore phytonutrient rich) but reduced in meat, refined grains, saturated fat, sugar and salt [122]. Many characteristics of the traditional Okinawan diet are shared with other healthy dietary patterns, including traditional Asian diets (especially Japanese), native Hawaiian (taro and sweet potatoes), Mediterranean (both vegetable heavy), and the researcher designed dietary approaches to stop hypertension (DASH) and portfolio (cholesterol lowering) diets [119]. Overall, the important shared features of these healthy dietary patterns include: high intake of unrefined carbohydrates, moderate protein intake with emphasis on vegetables and legumes, fish, and lean meats as sources, and a healthy fat profile (higher in mono/polyunsaturated fats, lower in saturated fat; rich in omega-3). Additionally, the lower caloric density and higher amounts of fiber from such plant-based diets results in lower caloric intake, a lower glycemic load and a higher intake of phytonutrients; all important for minimizing risk for age-associated disease (cardiovascular disease in particular) and maximizing odds for healthy aging and longevity. The oldest old in Okinawa may also have had a longevity dividend from mild caloric restriction and an unusually high amount of foods with potential CR-mimetic effects. These mostly shared dietary characteristics reduce the risk for chronic age-associated disease and promote healthy aging and longevity through multiple mechanisms, including reduced inflammation and oxidative stress.

Recent studies of humans who practice CR (up to 15 years) have shown adaptations consistent with the model organism data, that is, lower risk for CVD and cancer, and biomarker changes suggestive of slower aging [118]. The Okinawans have long been of interest to CR researchers because they may represent the best human example of a naturally calorically restricted population (with optimal nutrition) and the study of their lifespan, healthspan, mortality and morbidity patterns could provide important information on the long-term effects of CR in humans [120]. Some researchers have argued that the Okinawans achieved a long life expectancy for genetic (or other) reasons, but the rapid disappearance of the CR phenotype, as well as the longevity disadvantage in younger Okinawans who did not experience CR suggests otherwise [119]. Population-wide CR was over by the 1960s, and generations thereafter have had a higher BMI across all age strata, as well as more metabolic syndrome and worse cardiovascular risk factors than other Japanese. The life expectancy advantage for Okinawans, which used to be the highest in Japan for all ages, is now seen only in older ages, consistent with a residual CR-related cohort effect in older Okinawans [120].

In addition to CR, several nutritional factors are thought to be important for Okinawan longevity. For example, the health properties of particular foods from the traditional Okinawan diet may mimic the biological effects of CR, acting as caloric restriction ‘mimetics’. CR-mimetics are compounds that provide the physiological benefit of CR without the need for restriction of calories. The traditional Okinawan diet appears to be a rich source of CR-mimetics [119]. In the Okinawan language, the term *nuchi gusui*, literally means ‘food is medicine’ and reflects the cultural context wherein commonly consumed dietary items, including foods, herbs and spices are also used as folk medicines. Common items that play dual roles as both traditional medicines and foods include sweet potatoes (pulp, skin and leaves), bitter melon, multiple green leafy vegetables, ginger, turmeric, mugwort (*Artemisia vulgaris*), peppers (*Piper hancei*) and carotenoid-rich marine foods (such as seaweeds), among others [119].

We may ask why Okinawa appears to have so many food items with medicinal properties, but climate may offer a scientific basis for this phenomenon. Compounds that have potential CR-mimetic properties, such as carotenoids, flavonoids and other phytochemicals, are synthesized by plants to help scavenge free radicals formed due to stress from extremes of heat, cold, insects, UV light or other threats that are common to sub-tropical Okinawa. Murakami et al. [82] investigated typical food items from Okinawa and compared them to food items from mainland Japan and found that foods from Okinawa had, on average, stronger free radical scavenging properties. Of over one hundred food items tested for anti-oxidative and anti-nitrosative activity, many were shown to be promising anti-inflammatory agents, with implications for the prevention of inflammation-associated carcinogenesis, especially two kinds of turmeric, wild Okinawan turmeric (*Curcuma aromatica*) and Okinawan zedoary (*Curcuma zedoaria*); and ‘Botanbofu’ or ‘Sakuna’ as it is known in Okinawa (*Peucedanum japonicum*).

The most common compounds (polyphenols, flavonoids and similar compounds) can initiate a cellular stress response and induce beneficial molecular adaptations that are collectively known as ‘hormesis’ or a potentially important mechanism for the health-enhancing effects of caloric restriction [94]. Moreover, Davinelli et al. [27] proposed that phytohormetic stress resistance also involves the activation of nuclear factor erythroid 2-related factor 2 (Nrf2) signaling, a central regulator of the adaptive response to oxidative stress, which may partly account for CR’s anti-aging effects.

Hormetic phytochemicals have received much attention for their potential pro-longevity effects and ability to act as potent activators of sirtuins (upstream of FOXO3 (i.e., Forkhead Box O3, a protein coding gene, the deregulation of which is involved with tumorigenesis; and a variant is associated with longevity)), the most notable among them being resveratrol [75]. Interestingly, several of these compounds that are found in Okinawan plants, such as chebulagic acid (a resveratrol derivative) and sequiterpenoids, seem to be even more potent than resveratrol, at least in terms of some anti-aging properties, such as the ability to inhibit reactive oxygen species (ROS) and nitric oxide (NO) [85]. Many are also potent activators of FOXO3 transcription, a key transcription factor from the insulin-IGF-1 signaling pathway [29]. FOXO3 appears essential for caloric restriction to exert its beneficial effects [121, 123], and allelic variation in the FOXO3 gene is strongly associated with human longevity [121]. In fact, this finding has been replicated in multiple independent populations and is one of only two consistently replicated genes associated with human longevity [31]. Finally, and importantly, there are numerous promising food compounds from the traditional Okinawan diet that have been shown to modulate FOXO3 expression [119] and research is currently underway with the purpose of identifying the most promising candidates with therapeutic potential.

In summary, older Okinawans consumed a diet consistent with mild caloric restriction and rich in foods with CR-mimetic effects, including the staple sweet potato, marine-based carotenoid-rich foods, such as seaweeds, and a myriad of herbs and spices. A CR-like phenotype can be witnessed in older Okinawans which includes smaller stature, less age-associated disease, longer average and maximum lifespan and longer healthspan. Moreover, the epidemiological evidence supports a link between mild caloric restriction, CR-mimetic foods commonly consumed in the traditional diet, and the healthy aging phenomenon in Okinawa [120]. Biological pathways through which caloric restriction (and CR-mimetics) operate are currently being better characterized and the insulin-IGF-1 signaling (IIS) pathway appears to be critical. FOXO3 is a major regulatory gene in the IIS pathway and acts as a transcription factor that induces changes in gene expression in many downstream target genes in response to biological stress. It is activated by CR and CR-mimetic compounds commonly found in the traditional Okinawan diet. This may have profound implications for healthy aging and longevity.

## Systems approaches for defining the dietary landscape

Uptake and metabolism of food is a highly complex process and requires the coordinated interaction of cells, tissues and organs to establish homeostasis in multi-cellular organisms. Already minor disturbances in this interaction can cause disease or malfunction of the whole system. On the other hand, the metabolism has to be prepared to act on various fuel types and thus to convert various types of nutrients into energy, biomass, and at the same time to build up a storage pool for periods of fasting.

Systems approaches in the field of nutrition aim to understand the complex interplay of flux, metabolism, utilization, and regulation of nutrients from exogenous and endogenous origin in whole nutritional systems [69]. In this context, stable-isotopes are a valuable and non-invasive tool to profile the dynamics of metabolite turnover of (I) specific metabolic pathways of interest [24] and (II) on a whole organism scale [18, 52, 61]. In this contribution, we discuss the application and value of stable-isotope labeling in the field of nutrition.

To warrant the immediate and stable availability of energy even under high load conditions where complete carbohydrate oxidation is too slow or even not possible, the body must ensure that production and clearance of metabolites such as glucose, lactic acid or alanine are balanced. In humans and other mammals, the Cori cycle is a metabolic pathway that allows glucose homeostasis and involves the concerted action of central organs as it recycles alanine or lactic acid produced by glucose-consuming tissues back to glucose. Lactic acid and/or alanine produced in metabolically active tissues are transferred into the blood stream and transported to the liver. The liver takes up lactic acid and alanine, and recycles two of these molecules back to glucose in an energy-consuming fashion (gluconeogenesis). The so-assembled glucose is secreted back into the blood stream. Hepatic gluconeogenesis is, thus, essential for plasma glucose homeostasis, and when deregulated can be a cause for hyperglycemia, especially in type 2 diabetes mellitus [24].

The dynamics of gluconeogenesis in vivo can be revealed by oral administration of ^13^C-stable-isotope-labeled glucose and time-resolved blood sampling. We developed a protocol to extract polar metabolites from dried blood spots (DBS) and apply gas chromatography coupled with mass spectrometry (GC–MS) to acquire mass spectrometric data for plasma metabolites. To extract pure mass-spectra from these complex data, we apply the ion-chromatographic deconvolution algorithm of our MetaboliteDetector software [53]. Finally, based on the deconvoluted mass-spectra, we correct the compound spectra for naturally occurring stable-isotopes and determine mass isotopomer distributions (MIDs) for target metabolites. These time-resolved enrichment patterns in combination with absolute concentrations of the target metabolites both obtained from DBS sampling and GC–MS measurement are used as input for a simple mathematical ordinary differential equations (ODE) model. By solving the equation system, we are able to determine quantitative and robust values for glucose production (GP) and gluconeogenesis (GNG).

**Fig. 2:**
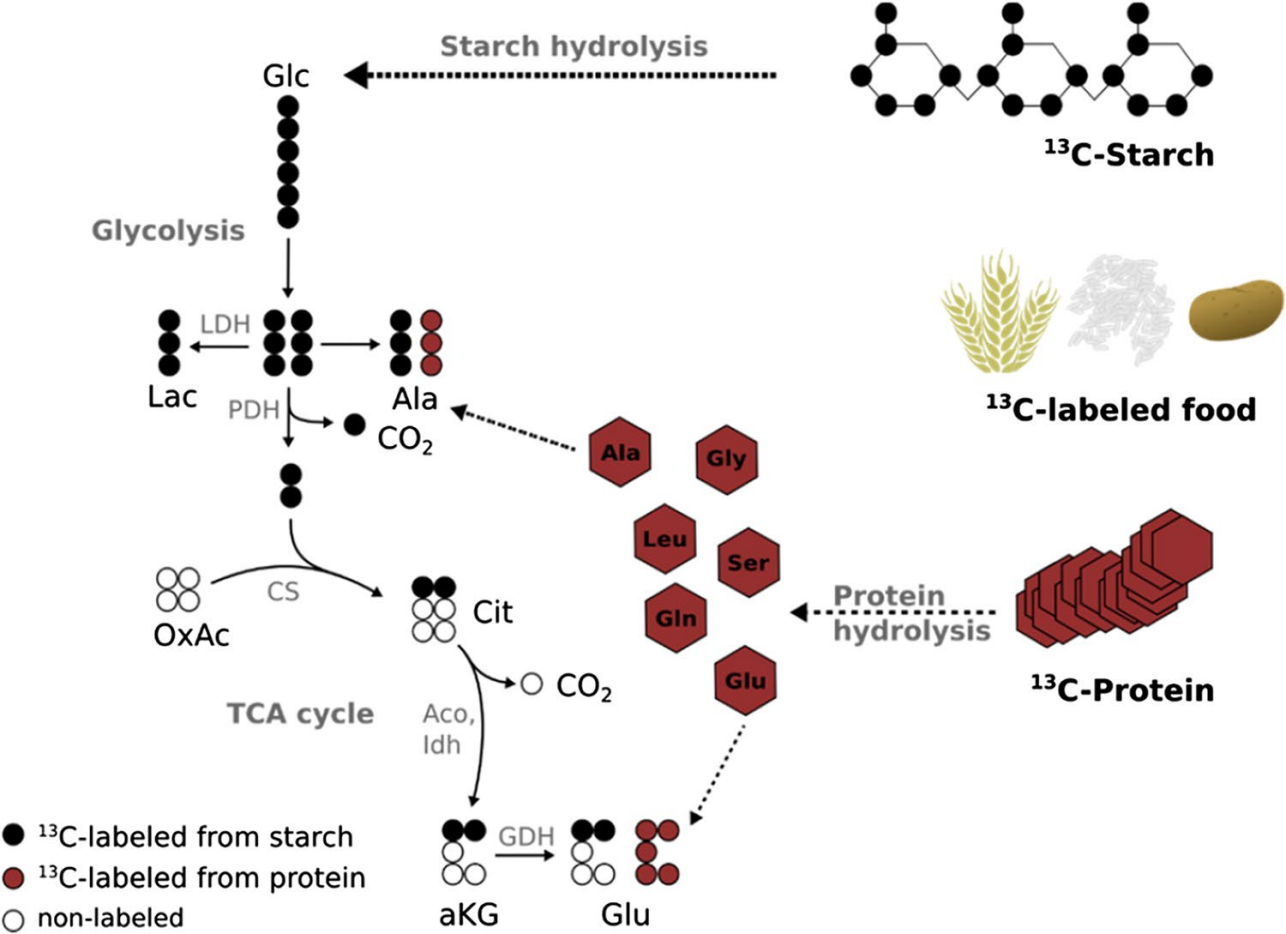
Fully ^13^C-labeled food products based on, e.g., wheat, rice or potato are composed of ^13^C-labeled starch and ^13^C-labeled protein. The labeled hydrolysis products, labeled glucose (*black*) and free labeled amino acids (*red*) enter metabolism and enrichment patterns in the plasma metabolites, measured by GC–MS. *Lac* lactate, *Ala* alanine, *OxAc* oxaloacetate, *Cit* citrate, *aKG* alpha-ketoglutarate, *Glu* glutamate, *LDH* lactate dehydrogenase, *PDH* pyruvate dehydrogenase, *CS* citrate synthase, *Aco* aconitase, *IDH* isocitrate dehydrogenase, *GDH* glutamate dehydrogenase

While already providing exact and quantitative metabolic turnover rates for metabolites directly involved in glucose metabolism, such a ‘small system’ does not highlight other parts of whole body homeostasis, as for example, metabolism of amino acids. To address this limitation, we are extending our focus to include quantitative flux data for more metabolites. For this purpose, we follow the fate of fully ^13^C-labeled food products after ingestion by human individuals. The application of labeled nutrients has been reported for various applications, e.g., the investigation of postprandial response [36], and is mostly based on food products from ^13^C-labeled plants like wheat, rice, potato or algae grown in a saturated ^13^CO_2_ atmosphere. Due to the fact that all ^12^C carbon atoms are substituted by ^13^C isotopes, every molecule has an increased molecular mass. In case of wheat and rice, the major molecule types are starch and protein. Therefore, the digestion and metabolism of these bio-polymers can be traced by time-resolved plasma sampling, subsequent GC–MS measurement, and determination of isotopic enrichment patterns of plasma metabolites of interest (Fig. 2).

As shown in Fig. 2, depending on the origin of the labeled metabolite, different mass isotopomers can be detected. For example, glutamate can be synthesized from starch hydrolysis via glucose, glycolysis, and TCA cycle metabolism and two labeled carbon atoms from glucose are conserved. In this case, the molecular mass of glutamate is increased by 2 U (M2 isotopologue). On the other hand, glutamate appears as a hydrolysis product of protein resulting in a fully labeled glutamate molecule. In this case, its molecular mass is increased by 5 U (M5 isotopologue). Combining these data with information of the composition of the applied food product, a biochemical pathway model can generate quantitative information on the dynamics of the postprandial response [36]. Such approaches to profile multifaceted systems result in the generation of high amounts of complex data. Therefore, computer-based tools and programs as well as high quality statistical analysis are increasingly important for deriving meaningful conclusions.

Nutrition is highly influencing our personal health, and it was recognized that dietary interventions in disease are important to support clinical therapy. Therefore, the generation of innovative systems approaches in the field of nutrition will be essential. As the analytical techniques and the computer tools improve, the extension of systems approaches by stable-isotope driven techniques adds invaluable information on the dynamical scale [52, 61].

## Bringing a ‘big data’ component to the wellness discussion

As healthcare becomes more data-rich and proactive, it will focus more on maintaining and enhancing wellness rather than just reacting to disease. The Institute for Systems Biology in Seattle (ISB) recently launched the large-scale ‘100K Wellness Project’ that integrates genomics, proteomics, transcriptomics, microbiomes, and clinical chemistries along with wearable devices to monitor wellness and disease. This project has begun with a proof-of-concept pilot study in a set of 108 individuals, the ‘Pioneer 100 Project,’ completed this past year. The interpretation of these data led the participants to actionable findings in the areas of medicine, exercise, and nutrition.

Current systems for dealing with health issues remain mostly reactive, with patients reporting to their clinic once symptoms are fully developed. This is a major impediment to the early diagnosis of complex diseases. Few physiological and biochemical parameters are measured routinely, and only after long intervals, so changes might not be identified until many months after the initial stages of disease. Furthermore, parameters vary greatly depending on time of day, gender, genetics, age, exercise schedule, environment, and many other factors. These sparse measurements are then typically compared to reference ranges determined from large population averages that may not be entirely relevant. As a result, it is very difficult to accurately determine if a significant health transition has occurred in a particular individual. Today, it is not generally possible to predict when we will become ill.

What if this were not the case? Imagine if one could learn how many years he or she were away from a specific illness. No one can avoid every risk factor for every disease, but much can be done if one knows what to prioritize. Good doctors always remind their patients to eat healthy and exercise. But what are the right foods? What are the right exercises? Only by engaging healthcare consumers as active participants who provide both health-related data and insights into pathophysiology can biomedical innovation accelerate.

This new scientific wellness strategy is described as personalized, predictive, preventive, and participatory (P4) medicine. The participatory component describes patients, researchers, physicians, and the entire healthcare community joining together to make health maintenance and the practice of medicine as highly proactive activities. Implementation requires two central activities. The first is the establishment for each individual of a ‘dense, dynamic data cloud’ consisting of billions of data points. Computational analysis of these data clouds will inform health decisions by providing actionable hypotheses chosen to optimize wellness and minimize disease. The second is patient-data integration that will uncover biological networks defining health, and that are perturbed in early disease stages. Through an understanding of these differences, fundamental insights will be gained [54].

A few highly motivated members of the nascent quantified self movement have started collecting their own data clouds. Larry Smarr (UC San Diego) is one such pioneer. After establishing solid baselines, he noticed an unexplained spike in his circulating C-reactive protein (CRP) levels—an indicator of inflammation. On sharing this with his physician, he was asked, ‘Do you have a symptom?’ and, when he answered in the negative, was sent away without treatment. Shortly after, he did indeed experience an acute, dangerous event that required urgent care [102]. Michael Snyder (Stanford University) is another. In his single test subject (not officially identified), he observed a number of distinct changes in blood biomarkers, reflecting pathways activated by viral infections and also by the onset of diabetes. Proteomic data indicated the onset of an elevated glucose response, following shortly after a respiratory syncytial virus infection [23]. Taken by itself, this could be mere coincidence, but once comparable data clouds are shared by thousands of individuals, real statistical analysis becomes possible.

The ultimate goal is to gather comprehensive genetic and physiological data, regularly, for a large number of people, over a long period of time. The objective is to observe all measurable changes that occur as individual transition between disease and healthy states and vice versa. Towards this goal, ISB recruited volunteers to the ‘Pioneer 100 Project’. This pilot project tested the feasibility of collecting and interpreting multifaceted data for multiple individuals, providing them with real-time feedback from the data, and coaching them on ways to improve their health and wellness.

ISB successfully enrolled 108 volunteers, or ‘Pioneers’, into an IRB-approved study led by Profs. Leroy Hood and Nathan Price. Pioneers ranged in age from their 20s to over 88 years and were approximately balanced between male and female. The study ran for 9 months in 2014. Each pioneer had their whole genome sequenced and provided blood, urine, saliva samples at 3-month intervals for metabolomic, proteomic, and additional chemical analysis. The gut microbiota were also surveyed three times by 16S rRNA sequencing. Participants engaged in continual self-tracking and lifestyle monitoring via digital pedometers and questionnaires. A registered dietitian served as a ‘wellness coach’ to share and interpret results with the participants. A physician reviewed all data before sharing [93].

Correlations between genetic predispositions and measurements were found for both simple traits and complex phenotypes. For example, by integrating previously identified genetic variants, each with a small effect on elevated LDL cholesterol risk, individualized risk scores were calculated. Increasing baseline LDL cholesterol levels were found to correlate with increasing genetic risk. Cohort-level statistics on all measurements also revealed that the largely ‘healthy’ volunteers had a high rate of initial lab results outside of normal range. Based on these results, all of the pioneers received actionable recommendations from their coaching phone calls. At the final time point, out-of-range measurements had dropped in diabetes, cardiovascular disease, inflammation, and nutritional categories. Two pioneers had genetic variants putting them at severe risk for hemochromatosis—a potentially life-threatening excess of blood iron. In fact, both had elevated iron and ferritin levels and neither were previously aware of this condition. By the end of the study, both had their iron levels under control and affected family members were made aware of their risk.

Even in a relatively small cohort of 108 individuals, the study uncovered proto-disease states, nutrient deficiencies, and unhealthy heavy metal levels. During the course of this study, the participants were thoroughly engaged by providing questionnaire data, biosamples for analysis, discussing the findings and the health recommendations with the wellness coach, and participating in pioneer social events. Pioneer feedback provided three consistent messages. First, participants understood that their genome does not control their health destiny. Second, the provided data enhanced their ability to take control of their health. Finally, many people are less healthy than they think—each pioneer had multiple actionable possibilities for enhancing their health.

## The role of supplements to support a healthy life

According to the WHO, globally, life expectancy is increasing in the majority of countries [126]. Living longer is not the issue anymore but living longer healthy and better is the challenge. The proportion of people aged over 60 years is growing faster than any other age group. Consequently, from 2000 until 2050, the proportion of the global population over 60 years will double from approximately 11 to 22%. This can be interpreted as a success story for public health policies and for socioeconomic development, but at the same time it also challenges societies to adapt and overcome the health and economic burden of aging and non-communicable diseases. ‘Healthy’ refers to physical, mental, and social well-being as indicated in the WHO definition of health [126, 127]. Lifelong health promotion is the opportunity, with a clear emphasis on the fundamental role of nutrition and micronutrients, to reduce risks of chronic conditions and disability, and to delay the onset of non-communicable diseases. This provides a trifold benefit: for the individual, for the society, and for healthcare system and costs [41, 100].

Most of the world’s population has inadequate intake of one or more of the essential vitamins and minerals. While vitamin and mineral deficiency, and inadequacy are often seen as problems in developing countries it is less recognized as an issue, also in developed countries [63]. Vitamins and minerals are cofactors for proteins and enzymes of metabolism or have other essential functions in our body. A varied and balanced diet should provide enough of all of the vitamins and minerals; however, an unbalanced diet provides calories but not enough of the vitamins and minerals. Insufficient vitamins and minerals result in a deficiency disease, while the consequences of inadequacy are more difficult to diagnose. The Triage theory by Dr. Bruce Ames posits that, because of recurrent shortages of vitamins and minerals during evolution, natural selection developed a strategic-rationing response to moderate shortages so that the scarce nutrient is preferentially retained by proteins that are essential for short-term survival and reproduction [6, 45]. In contrast, proteins needed for long-term health and to defend against the diseases associated with aging, are starved for vitamins, and thus are disabled. Since the damage from moderate deficiency is insidious, its importance for long-term health is not clinically apparent. Mechanistic, genetic, and epidemiological evidence suggests that the metabolic trade-off accelerates aging-associated malfunctions and diseases, such as cancer, cardiovascular disease, immune dysfunction, and cognitive decline [45].

The following example summarizes the benefit of essential nutrients for a healthy life which could result in appropriate changes, characterized by health and vitality of the individual, and a positive impact on healthcare and the attendant economic costs. Nutrition is a complex topic and so are its fields of application; nevertheless, a significant scientific and medical consensus exists as to the importance of an appropriate level of micronutrient intake throughout the life course to support growth, foster health, and prevent the onset of disease. Appropriate micronutrient intake (vitamins and minerals)—as part of a balanced diet and in combination with fortified food and supplements—encourages health and well-being [8, 43].

Micronutrient deficiencies and inadequate micronutrient intake compared to recommendations can have serious health consequences for individuals; they also have a wider impact on societies, economies, and healthcare and welfare systems. Healthcare costs are a significant part of the gross domestic product (GDP), expenditures have risen significantly; a 10% increase in non-communicable diseases results in a 0.5% reduction in annual economic growth [41, 100]. There is growing evidence from food intake surveys that a sufficient intake of micronutrients has not reached in many countries compared to the recommendations. Reasons for this are changes in lifestyle, different eating patterns with a higher share of outdoor eating and an increase of processed foods. As the insufficient intake does not result in immediate consequences for a person, the impact and long-term effects on health and well-being are often neglected [110].

A map indicating the vitamin D status in different countries and populations showed a surprisingly low vitamin D and initiated a call to act for evidence-based programs of vitamin D fortification of foods for the general population and/or supplementation in risk groups. An inadequate status in vitamin D impacts several body functions [57, 68, 113]. The classic role of vitamin D is connected to bone health: vitamin D is essential for the uptake and for the transportation of calcium, next to other factors like vitamin K. There are a number of emerging health benefits connected to an adequate vitamin D status, for example muscle strength and the risk of falling in advanced age; vitamin D strengthens the immune system and reduces the risk for multiple sclerosis. It also has an impact on the risk for cardiovascular diseases because it reduces high blood pressure, and it reduces the risk of some cancers as it inhibits or reduces cell proliferation. An analysis of published population-based studies in Europe, where the 25-hydroxy-vitamin D status was measured in blood, demonstrated for many countries a status below the desired level. There are different recommendations in place: the Institute of Medicine in the US recommends a status of 50 nmol/L or higher for the general population as optimal; the authorities in Europe, the International Osteoporosis Foundation, the Endocrine Society and other organizations recommend a status of 75 nmol/L or higher. A number of human studies indicate that a blood level of 75 nmol/L 25-hydoxy-vitamin D and higher is optimal for reducing the risk of bone fractures, improving muscle strength and reducing risk for diseases like diabetes, multiple sclerosis and some cancers. There is currently only one country in Europe which is in the desirable level of 75 nmol/L 25-hydoxy-vitamin D; this is Finland. Some countries are in the range between 50 and 75 nmol/L and the general population in countries, for example, Germany, Switzerland, and Italy are even below 50 nmol/L. For elderly, there are no data available which are representative for a complete country. An assessment for Germany has indicated that supplementation of elderly—assuming a risk reduction for fractures by 20%—would result in healthcare cost reduction of 580–780 million Euros every year [41, 68, 100].

Vitamin E is an essential nutrient; it is a powerful anti-oxidant and has been recognized as being vital for preserving the integrity of the cell membrane [86]. Vitamin E is increasingly reported to be related to protecting essential fatty acids from lipid peroxidation, cognitive function, reducing the risk of Alzheimer’s disease, and reducing the negative health implications of fatty liver disease. However, the intake of vitamin E is generally low across all regions worldwide; the population intakes for α-tocopherol and vitamin E are below the recommended 15 mg/day for men and women in the US as well as other Western countries, like Germany, the United Kingdom, and the Netherlands [92]. A minimum serum level of 12 µmol/L α-tocopherol is needed to avoid deficiencies in the human body. Furthermore, results from several observational, prospective studies suggest a serum tocopherol concentration of 30 µmol/L and above to have beneficial effects on human health in the field of cardiovascular disease, some types of cancer, and mortality. Additionally, data from the 2003–2006 National Health and Nutrition Examination Survey (NHANES) show mean α-tocopherol concentrations below the optimal concentration for the total population and non-supplement users. Apart from differences in α-tocopherol concentration between supplement and non-supplement users, a higher proportion of younger rather than older adults had suboptimal α-tocopherol concentrations. Therefore, despite low incidence of overt vitamin E deficiency many American adults have suboptimal α-tocopherol status even when supplementing their diet [86, 92].

There is a robust literature suggesting that the health benefits of the long chain omega-3 fatty acids, eicosapentaenoic acid (EPA, 20:5n3) and docosahexaenoic acid (DHA, 22:6n3) for human use. Blood levels of EPA and DHA vary across the globe, with most of the countries and regions of the world having levels that are considered as low to very low [37, 39]. The very low to low range of blood EPA and DHA may have implications for the individual as well as for the society because of higher risks for non-communicable diseases, in particular cardiovascular diseases and cognitive decline. A recent meta-analysis, to date, the most comprehensive quantitative analysis of its kind within the peer reviewed biomedical literature, showed a significant decrease of CHD risk in people with elevated TG and LDL cholesterol levels by supplementation with omega-3 fatty acids [3, 77, 89]. It is necessary to consume significantly more than 250 mg of omega-3 fatty acids per day to be classified as having a high EPA/DHA status, resulting in a blood level required to achieve cardiovascular benefits. This optimal level can be achieved by eating fatty fish rich in omega-3, like salmon or cod several times a week [89]. As many people cannot or will not have a diet heavy in marine foods, food supplements and fortified foods are the most convenient and cost effective way to ensure that the optimal level is achieved. The very low to low range of blood EPA and DHA levels may have implications on the global risk for chronic disease as well as healthcare costs, with a recent study finding that regular consumption of omega-3 supplements in the range of 1000 mg/day could save €12.9 billion a year in healthcare costs related to CVD-related events in the European Union alone [41, 68, 100].

The consensus concerning the value of appropriate micronutrient intake is based on a robust body of evidence and several powerful nutritional and economic models. It is, however, critical to develop models that address the complexities of micronutrient interventions.

The extensive scientific knowledge currently available needs to be translated into cost-effective, practical public health solutions. These may include fortification and/or supplementation. The health and economic benefits of fortification or supplementation with certain micronutrients, e.g., vitamin A (to reduce infant mortality), iodine (to reduce goiter), vitamin D (primarily against rickets), and folic acid (primarily to reduce neural tube defects), is clear. The economic return of fortification varies from 1:24 for vitamin A fortification; to 1:46 for multivitamins (Copenhagen Consensus); up to 1:200 for iodine fortification. Many countries in the world already have mandatory fortification programs in place involving these micronutrients. The absence of such programs (for example for folic acid and vitamin D), is an unacceptable gap in our public health system and should be scrutinized in the light of the overwhelming evidence of the proven benefits of fortification/supplementation programs involving these micronutrients [8, 38, 110].

The above points should receive the urgent attention of the public health, as well as the nutrition community, policy makers, patient organizations, health insurers and other stakeholders, and next steps should be taken to give a healthy diet a higher priority. The implementation of food fortification programs, the development of nutrient-rich, energy-balanced foods and supplementation programs are all rated as important steps towards a healthy life [38]. So, in summary, healthy nutrition and reduction of healthcare costs are realistic objectives. A healthy diet including food fortification and use of supplements providing all nutrients is more influential than genetic factors to decrease morbidity, to support quality of life and healthy aging. Successful examples demonstrate that people understand the importance of eating healthy, however, that realization requires support and education. In communities where integrated programs have taken place, dramatic improvements in quality of life, healthier life and lower healthcare costs are reported. Relevant stakeholders should act together to develop and implement programs for healthy nutritional solutions to provide all essential nutrients and improve the quality of life; solutions are available.

## Nutrition and healthy aging: towards the development of personalized interventions

In most countries, average human lifespan is increasing at approximately 2 years per decade. However, these additional life years are often burdened by poor health, this is not an inevitable consequence of aging. The aging process is plastic and there is good evidence that nutrition is a major determinant of how well we age [62] and of risk of age-related disease and disability [84]. The challenge is to improve eating patterns (and other lifestyle factors) to maximize healthy aging. However, conventional approaches to changing eating patterns have often produced modest improvements only [65], which are difficult to sustain. Additionally, because of optimistic bias, people are often resistant to dietary change.

The worldwide obesity epidemic attests to the difficulty in changing dietary choices in healthy ways. However, in recent years, there has been growing interest in the use of personalized nutrition (PN) approaches to help people adopt healthier eating patterns. The PN approach is predicated on the idea that individuals will be more motivated to make appropriate and sustained dietary changes if they perceive that the advice and support offered is ‘personalized’ and, therefore, more directly relevant to each individual.

The Food4Me PN intervention trial, tested the hypothesis that personalizing nutrition advice would produce bigger and more appropriate changes in eating patterns and markers of health [21]. This large-scale intervention study recruited 1609 adults (aged 18–79 years) across seven European countries and randomized them to (i) conventional dietary advice (control) or to PN advice based on: (ii) individual baseline diet; (iii) individual baseline diet plus phenotype; or (iv) individual baseline diet plus phenotype plus genotype. Participants were recruited via the internet using the Food4Me website (http://food4me.org/) [21, 22], the intervention was delivered via the web and by e-mail and the participants used this website to upload dietary and other data. Participants collected buccal swabs (for DNA extraction and genotyping) and blood samples (dried blood spots for metabolite assay) at home and posted them to the recruiting centers.

At 6-month follow-up, participants randomized to the PN arms of the intervention had bigger improvements in eating patterns than those in the conventional dietary advice (control) group but there was no evidence of added advantage of using phenotypic or genetic information to tailor the personalized dietary advice [22]. The Food4Me Study also showed the potential benefits of using an internet-based platform to recruit participants and to deliver PN across multiple countries. This offers opportunities for scaling-up intervention delivery and, potentially, for improved cost-effectiveness [20].

Lifestyle-based interventions are likely to be more effective if they are offered during significant life-stage transitions when participants may be more receptive to change. In respect of healthy aging, retirement is a major intervention opportunity which has been exploited in developing living, easting, activity and planning (LEAP), an internet-based platform designed to deliver personalized advice to help individuals improve diet, physical activity and social roles/connections and so age more healthily [87]. A recently completed pilot study shows that this intervention was well-received by participants in the retirement transition with high participant retention [66]. This was further evidence that internet (digital) approaches are effective in engaging participants and in delivering PN.

## Sarcopenic-obesity: a view from the inside

Modern lifestyle choices are leading to a plethora of metabolic conditions and phenotypes. Excess calorie intake, coupled with an ever increasing sedentary lifestyle has brought about an increase in overall adipose tissue mass, as well as adverse distribution. Modern imaging methods have helped to identify an ever increasing number of body composition sub-phenotypes, all of which appear to have distinct disease risk. One such phenotype is the so-called sarcopenic-obesity, with an aberrant muscle mass content and abnormal fat distribution. This phenotype, contrary to classical sarcopenia, which is especially observed in the older populations, appears to arise as a direct consequence of the current obesogenic environment that modern humans have created for themselves.

Sarcopenia describes the age-related gradual decline in muscle mass and function [96]. The consequences of sarcopenia are evident in the quality of life of many older subjects, leading to frailty, fractures, disability and mortality [76]. The etiology of sarcopenia is not fully understood, but includes physical inactivity, chronic diseases, inflammation, and poor nutrition. Currently, there is no universal method for determining sarcopenia; however, options include the use of the relative appendicular skeletal muscle mass; lowest two quintiles of relative muscle mass or ratios of appendicular fat-free mass or whole body fat-free mass to height squared or skeletal muscle/body weight × 100 [25]. The lack of techniques to directly measure ‘muscle mass’ has significantly limited the objective and reproducible assessments of sarcopenia. Most studies today tend to use dual-energy X-ray absorptiometry (DXA) or bioelectrical impedance (BIA), although neither measure ‘muscle quality’. Some researchers have suggested the use of specific muscles such as the psoas or masseter muscles as potential markers of sarcopenia [59, 116], but these have not been fully validated.

In many instances of sarcopenia, reduced muscle mass is associated with increased fat mass, a condition known as sarcopenic-obesity [10]. Sarcopenic-obesity, has been described as ‘the confluence of two epidemics’ [97], with a combination of reduced muscle mass coupled with significant metabolic dysregulation. The reported prevalence of sarcopenic-obesity varies widely, ranging from 3.6 to 94% [9]. Moreover, despite the fact that in many cases the apparent ‘volume of muscle’ may appear unchanged or even increased, there is an additional burden of obesity in the elderly as decline in physical function reduces muscle quality [114]. Recently, it has been proposed that magnetic resonance imaging (MRI) techniques could play a significant role for a more objective diagnosis of sarcopenic-obesity [81], as they allow full body composition analysis during a single examination, including ectopic fat depots.

**Fig. 3:**
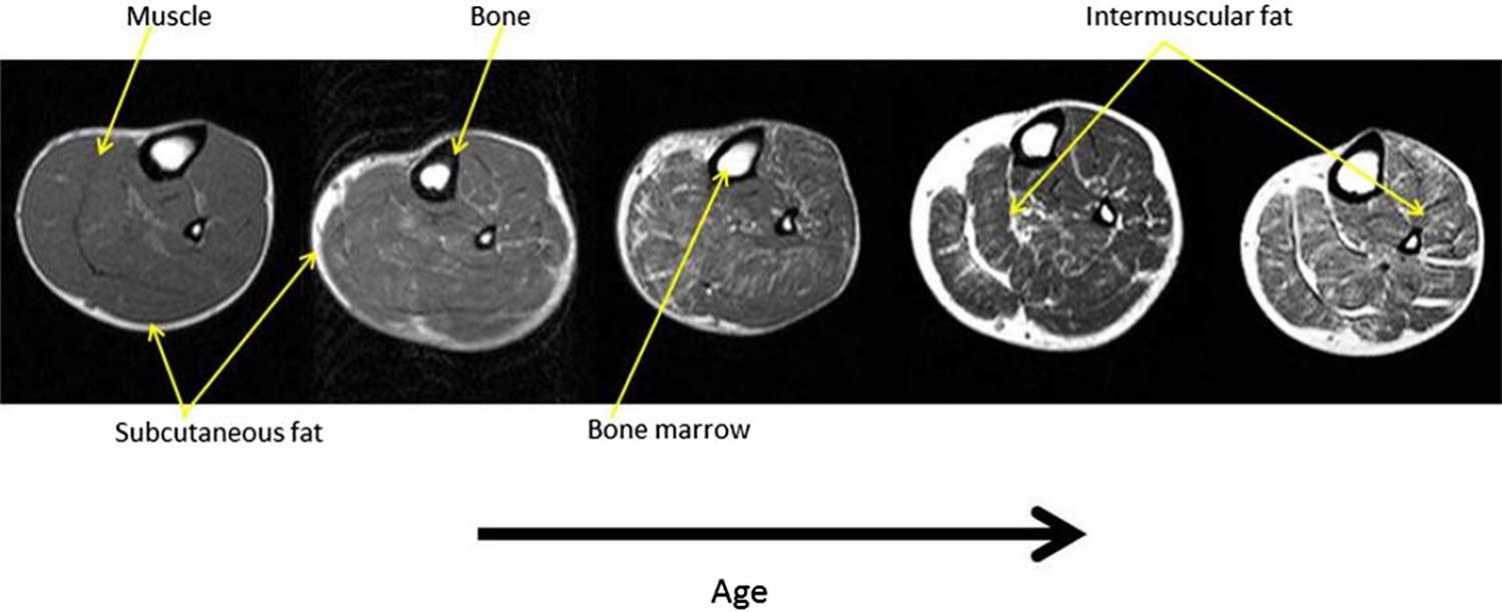
Axial MR images from the lower leg of 5 volunteers, showing typical changes arising as a result of the normal aging process. This includes both a reduction in the proportion of ‘muscle mass’ and the ‘quality’ of the muscle, with a visible increase in the amount of intermuscular fat deposited

Indeed, recent studies suggest that the sarcopenic-obesity phenotype should include ectopic fat depots such as the liver, pancreas and muscle. Both intra- (within muscle cells) and extra- (between muscle cells) myocellular fat have been shown to increase as a consequence of aging, inactivity, obesity and poor dietary choices [72, 109]. This, in turn, may underpin the deterioration in ’muscle quality’ observed with this phenotype. This is clearly illustrated in Fig. 3, where increasing fat infiltration across muscle groups can be observed with aging, in the absence of changes in ‘muscle volume’. Thus, understanding the contribution of ectopic fat accumulation may be particularly relevant to sarcopenic-obesity since there is a significant association between intermuscular fat accumulation and decline in gait speed, independent of thigh area [13]. This suggests that measurements of muscle quality/composition may be pivotal.

In conclusion, our current obesogenic environment, characterized by access to calorie rich foods coupled to uncontrolled levels of inactivity, is leading to the propagation of a sarcopenic-obesity phenotype across the population and across the generations. The advent of sophisticated high-throughput imaging systems are allowing the development of a more detailed and reproducible definition and measurement of sarcopenic-obesity, thus, determining its impact on health.

## Plant-based dietary patterns, chronic disease risk, and healthy longevity: an emerging paradigm for phytonutrients and health promotion

Over the past two decades, epidemiological studies have shown a consistent inverse relationship between fruit and vegetable intake and risk for chronic disease, and are the basis for the WHO recommendation for adults to consume at least 400 g/day fruit and vegetables. In the same timeframe, other epidemiological research has identified dietary patterns featuring an abundance and variety of plant foods as a common characteristic in populations that experience exceptionally healthy longevity. The Okinawan and Mediterranean style diets are two well-known examples (see “Dietary patterns and longevity: insights from the oldest old in Okinawa”, this article). As these two bodies of research have developed, rapid advances have been made in understanding the biology of aging. This emerging research suggests that the age-related decline in cells, tissues and organs underlies the onset of many chronic diseases. Studies in animal models suggest that interventions that slow the rate of aging hold potential to both reduce the risk for chronic disease, and also promote health. The provocative potential connections between these areas of research have been the basis for science at the bench, as well as in the clinic, to characterize the composition, assimilation and metabolism of plant food nutrient components, and their effects on biological mechanisms of aging.

Two prominent examples of science focused on the role of plant-based dietary patterns for reducing and managing chronic diseases come from Wang et al. [117] and Boeing et al. [17]. Wang et al. demonstrated an inverse relationship between the hazard ratio for all-cause mortality and fruit and vegetable consumption. Higher consumption of fruits and vegetables was significantly associated with a lower risk of all-cause mortality. There was a threshold around five servings of fruits and vegetables a day, after which the risk of all-cause mortality did not reduce further. Boeing et al. summarized the strength of evidence behind fruit and vegetable intake and specific chronic diseases. The conclusions from this work suggest that increased fruit and vegetable intake is likely protective against cardiovascular disease and cancer, and also suggest a protective role against other chronic diseases that are less often associated with mortality.

A new paradigm for plant-based dietary patterns, phytonutrients, and health promotion comes from a selection of literature focused on four emerging areas of science: (i) plant-based dietary patterns in cohorts experiencing healthy longevity, (ii) plant-based diets and wellness outcomes, (iii) advances in understanding the biology of aging, and (iv) phytonutrient interactions with aging mechanisms.

Selected Okinawan and Mediterranean populations have been documented to experience unusually healthy longevity. Interestingly, these populations have plant-based dietary patterns that are well studied in terms of their protective effects against chronic disease. These populations not just survive to old age, they do so with high quality health until late in life. More recently, the National Geographic Society has sponsored a body of ethnographic research that features a selected few demographic groups around the world, all of which experience unusually healthy longevity similar to the Okinawan and Mediterranean populations. These have collectively been named ‘Blue Zones’. Interestingly, plant-based dietary patterns rich in fruits, vegetables, whole grains, and legumes are a feature of the Blue Zones, in addition to active lifestyle, and other health promoting behaviors.

Emerging wellness outcomes research in humans suggests that plant foods hold potential to promote health and function of different organ systems. Green leafy vegetable consumption has been associated with improvements of heart rate variability measures, which may reduce the risk of cardiovascular disease through these favorable changes in cardiac autonomic function [91]. Strong inverse associations were found between plant-based anthocyanin intake and age-related decline in lung function as determined by forced vital capacity [74]. There is evidence that increased fruit and vegetable intake has a positive impact on immune function as determined by antibody response to vaccination [45] as well as an inverse association with 4-year weight change [14]. Vegetables having both higher fiber and lower glycemic load were more strongly inversely associated with weight change compared to lower-fiber, higher-glycemic-load vegetables. Intake of fruits, vegetables, fish, whole grains, legumes and dairy products is associated with improved bone mineral density [28].

A developing body of evidence in in vitro and animal model systems has identified several mechanisms that are thought to play important roles in aging. The primary mechanistic pathways result in reduced oxidative stress, suppressed low-grade chronic inflammation and induction of autophagy [101]. We have explored botanical extracts for their ability to influence one or more of these pathways. We evaluated the effects of turmeric, quercetin, and rosemary extracts on the activation of the nuclear erythroid 2-related factor 2–anti-oxidant responsive element (Nrf2–ARE) signaling pathway. Alone, each of these botanicals had modest Nrf2 activation. When combined, however, there was a surprising synergistic activation. This result is not only an example of the ability of selected phytonutrients to influence aging mechanisms favorably, but also an illustration of the potential for them to interact within a given pathway [79].

**Fig. 4:**
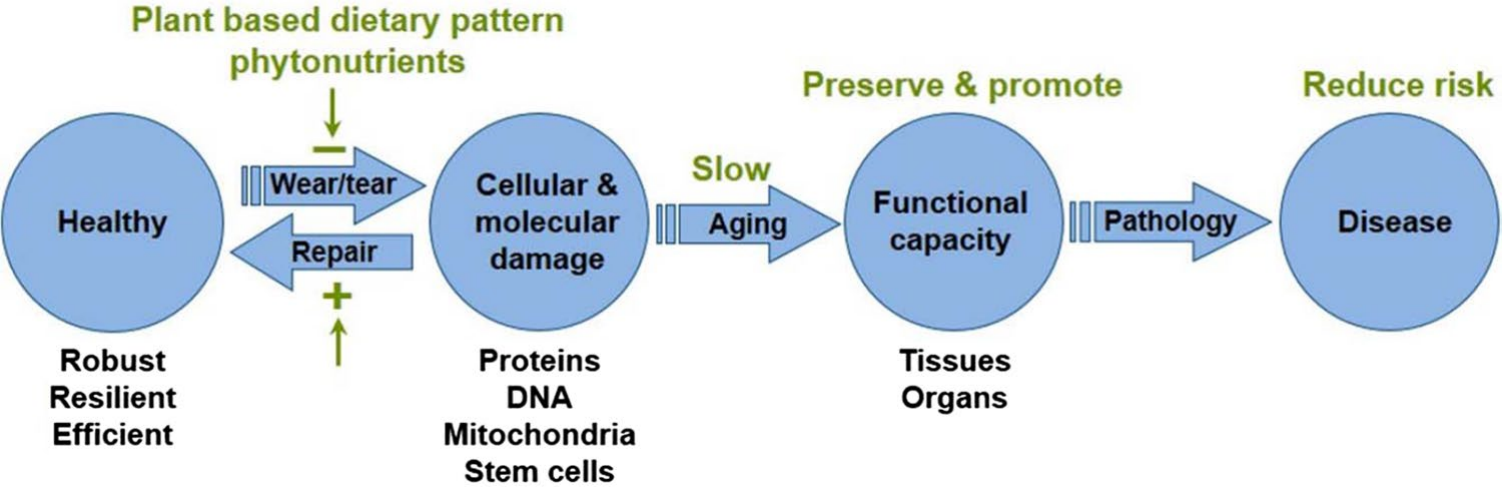
Schematic of current working models for the relationships between mechanisms of aging, age-related functional decline and chronic disease. The proposed interactions between these mechanisms and phytonutrients are depicted by *green text*

Advances in understanding the biology of aging suggest that the origins of chronic disease may lie in the age-related decline in cell, tissue and organ function. The schematic shown in Fig. 4 depicts a synthesis of current working models for the relationship between mechanisms of aging, age-related functional decline and chronic disease. Early in life, the majority of cells and tissues in the body reside at the far left hand side of this schematic. With the passage of time, however, the number of cells and tissues that exhibit compromised function rises such that there is a decline in functional capacity at the tissue and organ level. This process proceeds, if unimpeded, to pathology and the manifestation of disease.

Phytonutrients interact with aging mechanisms such that they hold potential to confer healthy longevity. Figure 4 depicts some proposed areas where phytonutrients conceivably can influence the rate of aging, but also promote functional capacity and reduce the risk for chronic disease. This is one rendering of the emergent paradigm for the role of diet, specifically phytonutrients, to favorably influence all of these outcomes.

Evaluation of the evidence as a whole supports the role of plant-based dietary patterns in promoting healthy longevity and suggests that there is an opportunity to intercept individual health trajectories with a healthy plant-based dietary pattern, with the result being improved health and function throughout life. There are, however, important considerations in this paradigm. First, phytonutrients are not likely to act alone as individual compounds, but as a collective family of nutrient compounds. Second, dietary patterns are more powerful lenses with which one can examine healthy longevity with respect to individual foods. No single food is likely to exert healthy longevity. Third, physical activity is most likely a large influencing factor for healthy longevity. Fourth, the gastrointestinal microbiota undoubtedly play a role in the assimilation of dietary components, as well as in the manifestation of end-physiological effects.

## Defining optimum nutrition: dynamic biomarkers to capture the complexity of health

Notwithstanding an increasing interest in specific diets to manage, reverse or even cure disease, the primary goal of healthy nutrition is to maintain optimal health and to prevent disorders [15, 44, 51]. Although this might only seem like a marginal difference, starting from the perspective of health, instead of disease, requires different ways of thinking and new approaches. Interestingly, many aspects of modern ‘healthcare’ are rather forms of ‘disease management’. It is perfectly understandable that this situation has evolved since health is intangible, variable, and difficult to predict. At the same time, and irrespective of its precise definition, there is general consensus that optimal health not only constitutes one of the most important values in our life, but also is a major determinant of social and economic stability. It is also clear that diet is one of the main lifestyle factors determining physical and even mental health. However, faced with difficulties to capture the effects of diet and nutritional interventions and their multifactorial character, many clinicians and policy makers struggle with the practical impact and feasibility of dietary measures. Effects of nutrition are subtle, complex and generally occurring very slowly. This complexity not only poses important scientific questions, but also fuels ongoing discussions, trends, and hypes in the field of nutrition and health. To address the knowledge gaps, several initiatives involving academic and industrial partners have been launched to develop new views and methodologies. Their focus is not only on measuring health effects, but also on trial design including the role of randomized controlled trials (RCTs), statistics, and methodology to accurately measure dietary intake. These developments are supported by the rapid progress made in -omics technologies, data management, statistics, and micro-electronics. Biomarkers for health are key in this process.

In many ways, modern medicine and nutrition science have drifted apart. Medicine has developed into a predominantly disease-oriented discipline, and in its wake the ‘one disease—one target—one drug paradigm’ has become a dominating principle. Understandably, this has consequences for thinking, training, and the way of doing research. In this respect the RCT is the golden standard to, for example, demonstrate the efficacy of therapeutic drugs. However, it is clear that nutrients behave differently from medicinal compounds. Unlike most medicines, single nutrients are involved in numerous endogenous processes and part of complex molecular networks regulating their availability, formation and degradation. As a result, their dose (or concentration) response curves, often U-shaped, are generally composed of multiple effect relationships. Acute effects or ‘quick wins’ are far less obvious than with pharmaceuticals. Another important point is that RCTs have often little usefulness or may not even be possible when studying the effect of diet and (or) specific nutrients [15, 42, 51]. To understand and quantify the way nutrition acts in improving, stabilizing or restoring health, better working definitions of ‘health’ were needed that take into account it’s dynamic, multi-dimensional and time-dependent character. These definitions have in common the ability to continuously adapt in varying circumstances. An example is ‘the ability to adapt and self-manage in the face of social, physical, and emotional challenges’ [55]. This paradigm dates back to the classical physiological, or even older principles of homeostasis. Nutrition plays important roles in maintaining or even strengthening a proper physiological bandwidth or flexibility (resilience). This has also been called ‘phenotypic flexibility’, being the resultant of the individual’s genotype, his/her physiological and psychological state at a particular point it time, his/her microbiota, etc. [112, 115]. Within this context, the (often gradual) onset of disease starts when and where adaptive processes fail. To measure these dynamic processes, a ‘systems approach’ is used, meaning that multiple biomarkers, preferably of different integration levels (e.g., gene, protein, metabolite, but also a physiological response, images, etc.) are analyzed at different time points and integrated into models [11]. Such combinations of health biomarkers are often different from the classical disease biomarkers. Furthermore, ‘stress’ or ‘challenge’ tests are used to measure the flexibility and resilience of health. Such tests measure the response to a metabolic, physical, psychological or immunological stressor. Different biochemical, physiological or psychological endpoints are in use, which are indicative of specific processes [104]. For example, a metabolic challenge test measures the response to a standardized meal or beverage that provides a carbohydrate or fat ‘load’ [60, 104, 125]. Next to metabolic stress tests, a laboratory can also apply physical challenge tests [58]. These tests, applying a strenuous exercise protocol on a bicycle ergometer, generate effects on immune function and intestinal permeability, which in turn can be used to investigate potentially positive effects of dietary interventions, probiotics, etc. Other challenge tests apply vaccination, experimental infection [106] or psychological stress [73, 98].

Although these concepts are increasingly acknowledged as the way forward in nutritional science, they have not made life easier in every respect. Their development is often hampered by complexity and costs. Inter-individual differences in responses can be quite large, and standardization and data exchange between labs is not always easy. Another disadvantage is that these tests usually only provide information on the resilience of a limited domain of health (improvement) indicators such as metabolic health, intestinal health, immune health, etc. Last but not least, their extrapolation to perceivable consumer benefits often remains difficult.

An interesting development will be the possibility to move challenge test approaches out of the conventional lab environment. Thanks to the rapid developments in ‘wearable’ technologies for continuous collection of health parameters [64], it is anticipated that a new generation of challenge tests can be developed and incorporated into systems for total health monitoring with ‘real life’ challenges. Provided that technical problems with data integration will be solved, such methodology would also enable further interdisciplinary integration, for example between physiology, epidemiology, geo-informatics, microbiology, consumer behavior, etc.

The generally subtle effects of diet and nutrition on health require methods and biomarkers that capture the dynamics and complexity of physiological resilience. Technological developments are moving extremely fast, offering exciting new ways to measure systems flexibility under real life conditions. Although still many technological and IT challenges lie ahead [2, 49] these ‘living lab’ approaches are likely to offer new ways for nutrition-related health optimization, self-empowerment, and to develop new products and services.

## Conclusion

The CRN-International Scientific Symposium and this article focus on ‘Optimal Nutrition,’ a subject that is nebulous and fraught with a myriad of definitions, boundaries, caveats, and nuances. The ten recognized experts offered their personal perspectives on this most challenging theme. Definitionally, ‘optimal’ would be deemed the ‘most desirable or satisfactory, most favorable, most effective’ and would be the ‘selection of a best element (with regard to some criterion) from some set of available alternatives’, and ‘nutrition’ would be the ‘act or process of nourishing or being nourished; specifically; the sum of the processes by which an animal or plant takes in and utilizes food substances’. However, even when seeking the most desirable process of nourishment, one must then consider that what may be optimal for one person or one region, may not be translatable across the spectrum of human idiosyncrasies.

Historically, the presence or absence of a disease was the measure of how poorly or how successfully one was entering older age. However, that concept is being reevaluated and redefined and ‘functional ability’ is becoming the new rubric. A combination of the intrinsic capacity (physical and mental) that an individual can rely on, relevant environmental factors, and the interactions between the individual and these characteristics is how WHO recommendsdefining ‘healthy aging’ [12, 88]. A geriatric population that is characterized by high prevalence of ‘frailty’, i.e., a progressive age-related deterioration in physiological systems that result in extreme vulnerability to stressors that in turn increase the risk of multiple adverse outcomes, to the individual as well as to the personal and public healthcare systems through increased dependence, has the potential for extreme personal, familial, governmental and societal costs in time and money. In the twenty-first century, most people can expect to live well into their 60s and 70s (and even older). In 2015, the WHO estimated that global life expectancy at birth was 71.4 years (http://www.who.int/gho/mortality_burden_disease/life_tables/situation_trends/en/). However, the quality of the growing number of added years of life expectancy is highly dependent upon and affected, for better or worse, by each individual’s ‘health’.

This year’s proceedings article offers insights into systems approaches, tracking and analyzing ‘big data’, use of key nutritional and dietary supplements, personalized interventions, dealing with obesity (visible and invisible), use of dynamic biomarkers and other perspectives. It is not the ultimate answer or definitive roadmap; however, it is highlighting opportunities and setting the stage for more robust discussion and broader and deeper recommendations.
